# Management of sunflower charcoal-rot and maize late-wilt diseases using the aqueous extract of vermicompost (vermitea) and environmental-safe biochar derivative (wood vinegar)

**DOI:** 10.1038/s41598-023-43974-2

**Published:** 2023-10-13

**Authors:** Osama M. Darwesh, Ibrahim E. Elshahawy

**Affiliations:** 1 Agricultural Microbiology Department, National Research Centre, Cairo, 12622 Egypt; 2 Plant Pathology Department, National Research Centre, Cairo, 12622 Egypt

**Keywords:** Biotechnology, Microbiology, Plant sciences

## Abstract

In Egypt, sunflower charcoal-rot caused by *Macrophomina phaseolina* and maize late-wilt caused by *Magnaporthiopsis maydis* are the most prevalent, and can lead to huge yield losses of both crops under epidemic conditions. In this study, the potential use of vermitea and wood vinegar for management of both diseases was investigated. Data revealed that, among the 17 bacterial strains obtained from vermitea, three strains named VCB-2, VCB-7 and VCB-11 were chosen for having the greatest in vitro inhibitory effect against *M. phaseolina* and *M. maydis*, with fungal inhibition values of 54.2; 61.7, 65.2; 74.0 and 57.1; 87.0% against both pathogens, respectively. These strains were identified as *Bacillus amyloliquefaciens*, *Serratia marcescens* and *Bacillus velezensis*, respectively. Wood vinegar significantly reduced the colony diameter of *M. phaseolina* and *M. maydis* in in vitro trials conducted on potato dextrose agar medium amended with the desired concentrations of 0.5, 1.0, 1.5, 2.0, and 2.5%. The efficiency increased with increasing wood vinegar concentration, and 2.0% was the most effective (100% suppression). Data from greenhouse experiments showed that the application of vermitea or wood vinegar tended to decrease the incidence (% dead plants) of sunflower charcoal-rot (by 61.1 and 66.7%) and maize late-wilt (by 70.6%). These treatments had positive impacts on the plant growth parameters, photosynthetic pigments and antioxidative enzymes of sunflower and maize plants. Data from field experiments showed that the application of vermitea or wood vinegar decreased the incidence of charcoal-rot (by 72.8 and 72.0%) and late-wilt (by 88.7 and 87.0%) as well as increased the production sunflower and maize plants.

## Introduction

Sunflower (*Helianthus annuus* L.) as the member of *Asteraceae* family is a significant global oil seed crop. It is a short-season crop (90–100 days) that can be grown twice a year, and there are significant efforts underway to expand the area under cultivation in order to increase local production of edible oil in Egypt. Unfortunately, several diseases, particularly charcoal rot disease, have a negative impact on sunflower production in Egypt^1,2^. Charcoal-rot is caused by the fungus *Macrophomina phaseolina* (Tassi) Goid (the pecnidial stage of *Scletroium bataticola* Taub.), which has a host range of more than 500 plant species^3^. Symptoms of charcoal-rot typically appear at the end of the growing season, after flowering^3^. Stunting, chlorosis and wilting are common symptoms^2^. It also causes lower yield and early senescence^4^. The development of microsclerotia at the base of the stems, which make them appear grey, is what is referred to as "charcoal-rot"^5^. The main source of inoculum is microsclerotia, which serve as survival structures and can germinate near roots during 2 days. However, germination can continue throughout the growing season. Root infection occurs very early in the season, but the factors influencing root colonization rate are not fully understood^6^. *Macrophomina phaseolina* infects and stays in plant tissue at the seedling stage. When conditions are favorable for disease development, which is typically when additional stress on the plant increases susceptibility to the pathogen, symptoms of charcoal-rot was appeared^7^.

On another way, maize (*Zea mays* L.) is considered the most significant cereal crops in Egypt. The most devastating or upsetting maize disease, late-wilt, which is caused by the soil-borne fungus *Magnaporthiopsis maydis* (also known as *Cephalosporium maydis* (Samra, Sabet, and Hingorani) and *Harpophora maydis*), results in significant yield and quality losses^8,9^. About 50 to 60 days after sowing, the plant begins to flower, at which point the first symptoms appear. The first signs are sudden wilting of nearby leaves, which advance upward over the following 2 weeks^10^. As the disease worsens, the leaves gradually lose their color and become dehydrated. Reddish-brown to yellowish streaks may be seen on the lower internode. The lower stem has dried out by that time, especially at the internodes, and looks hollow and shrunken. Later, *M. maydis* colonizes the entire stalk, suffocating the water supply and causing wilting by blocking the vascular tissue with hyphae and deposits that resemble gum. Secondary pathogen infection is frequently linked to late-wilt, which worsens the symptoms of the stem. Less ear growth occurs, and when it does, the kernels that do form are frequently damaged and immature and may harbor the pathogen. Disease severity is negatively correlated with grain supply and quality. These harmful processes may ultimately cause the plant’s death^11^.

Different disease management strategies have been put into practice to fight and eradicate sunflower charcoal-rot and maize late-wilt. Among them are cultural, physical, and biological techniques^12–15^. All of these techniques affect when used beforehand as a preventative measure^16^. As soon as a disease appeared, these techniques are no longer useful or effective. In that situation, chemical control is a good option for disease control. Chemical pesticides have been around for a while, and they offer quick, efficient, and affordable disease management^17^. The use of chemicals in agriculture has recently been shown to be less advantageous than previously believed. Both the person applying the chemical and the person using the treated material are at serious health risk. In addition to the target organism, pesticides also kill a number of beneficial organisms. Also, chemical pesticides continue to exist in toxic form in the soil, which pollutes the entire environment. Byproducts have taken a significant step in place of systemic materials due to humankind's growing awareness of its impact on ecosystems and the environment^18^.

Vermicompost (also known as organic fertilizer) is a byproduct of farmyard manure digested by the earthworm *Eisenia foetida* and the microorganisms that live within it. It has been widely used in organic agriculture not only for its beneficial effects on soil quality, but also for its remarkable ability to inhibit plant pathogens and promote plant growth^19^. Vermitea is a liquid vermicompost solution made by combining vermicast with water and fermenting it for a set period of time^20^. There are two kinds of vermitea: aerated and non-aerated vermitea. Nutrients and microorganisms are extracted during the steeping of vermicompost in water. The presence of microorganisms converts insoluble nutrients into soluble forms, which in turn promote a diverse range of organisms in vermitea during the brewing process. Both methods (aerated and non-aerated) of producing vermitea involve brewing matured vermicompost in water for a specific period of time and require filtration before application to plants^21^. Vermitea treatments have been shown to inhibit or kill a variety of fungi, parasitic nematodes, and other pests^22,23^.

A reddish-brown condensate liquid byproduct of the fresh wood gas combustion in an airless environment is called "wood vinegar," as one of biochar derivatives also known as "pyroligneous acid". Acetic acid, methanol, acetone, wood oils, and tars are all present in the liquid that is formed when the gas cools. It is widely used as a wood preservative, in health care products, and in agriculture. Liquid smoke, liquid wood, liquid vinegar, pyrolysis oil, pyrolysis liquid, wood liquid, wood acid, bio-oil, bio-crude oil, and wood distillate are synonyms for the wood vinegar^24^. Several substances, including hydroxy aldehydes, hydroxy ketones, sugars, carboxylic acid, and phenolic acid, are present in wood vinegar^25^. Recently, it has been used in agriculture (as a weed-control agent, anti-bacterial, and anti-fungal agent), the food industry, pharmaceuticals, and healthcare^26,27^. It is a cheap, all-natural product that doesn't harm living things in the environment^28^. According to the study of Matnork et al.^29^, it inhibits a number of fungal plant pathogens. However, no studies on the antifungal activity of byproducts (virmitea or wood vinegar) against sunflower charcoal-rot and maize late-wilt have been published. So, the goal of the current study was to estimate the effectiveness of vermitea and wood vinegar against *M. phaseolina*-caused charcoal-rot of sunflower and *M. maydis*-causes late-wilt of maize in vitro, in pots and field trials.

## Results

### Laboratory trials

#### Antagonistic activity of vermitea bacteria

**Figure 1:**
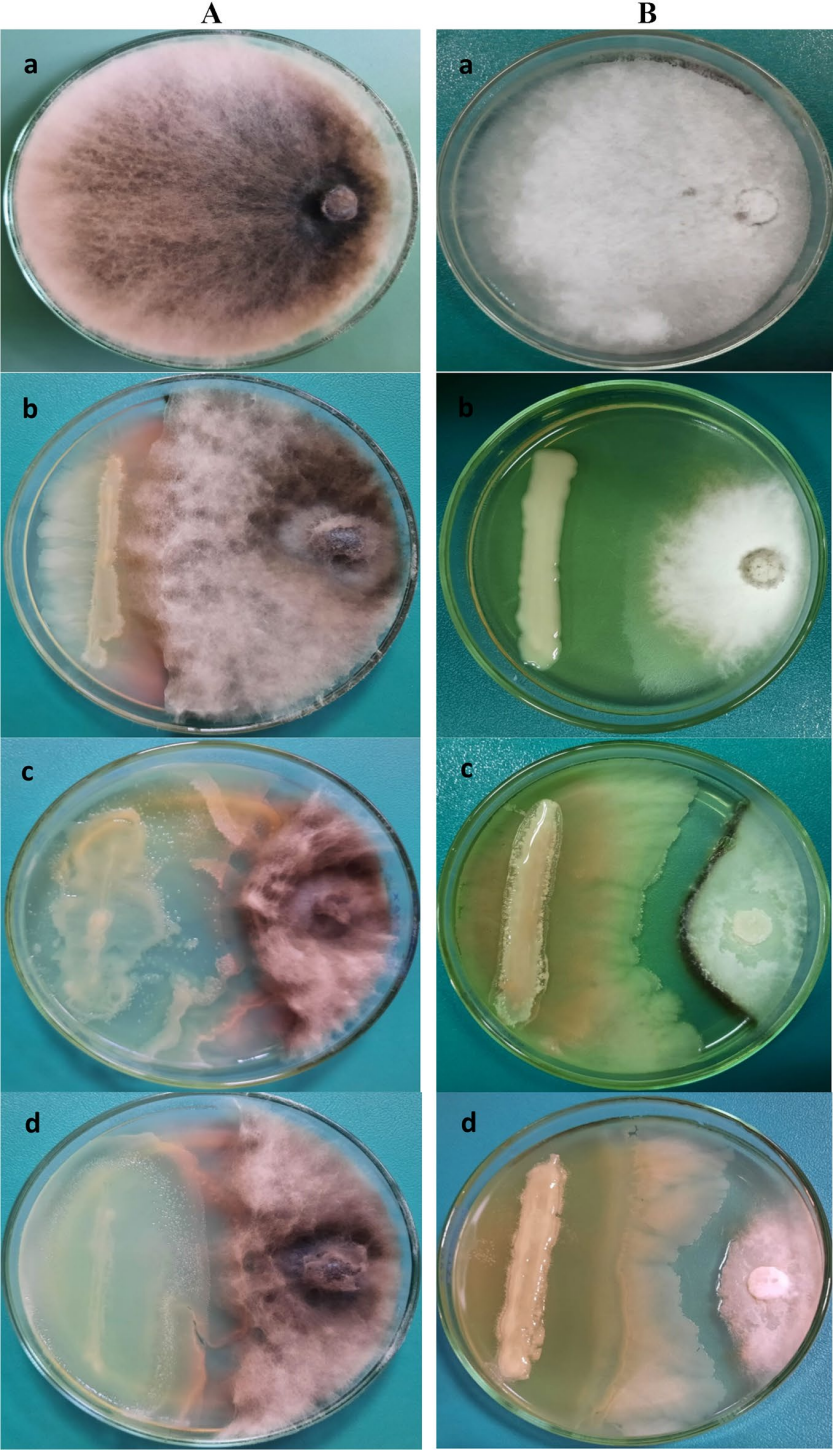
In vitro antagonistic activity of vermtea bacteria against *M. phaseolina* (**A**) and *M. maydis* (**B**) after incubation at 27 ± 2 °C for 7 days. Control (**a**), *Bacillus amyloliquefaciens* (**b**), *Serratia marcescens* (**c**) and *B. velezensis* (**d**).

**Figure 2:**
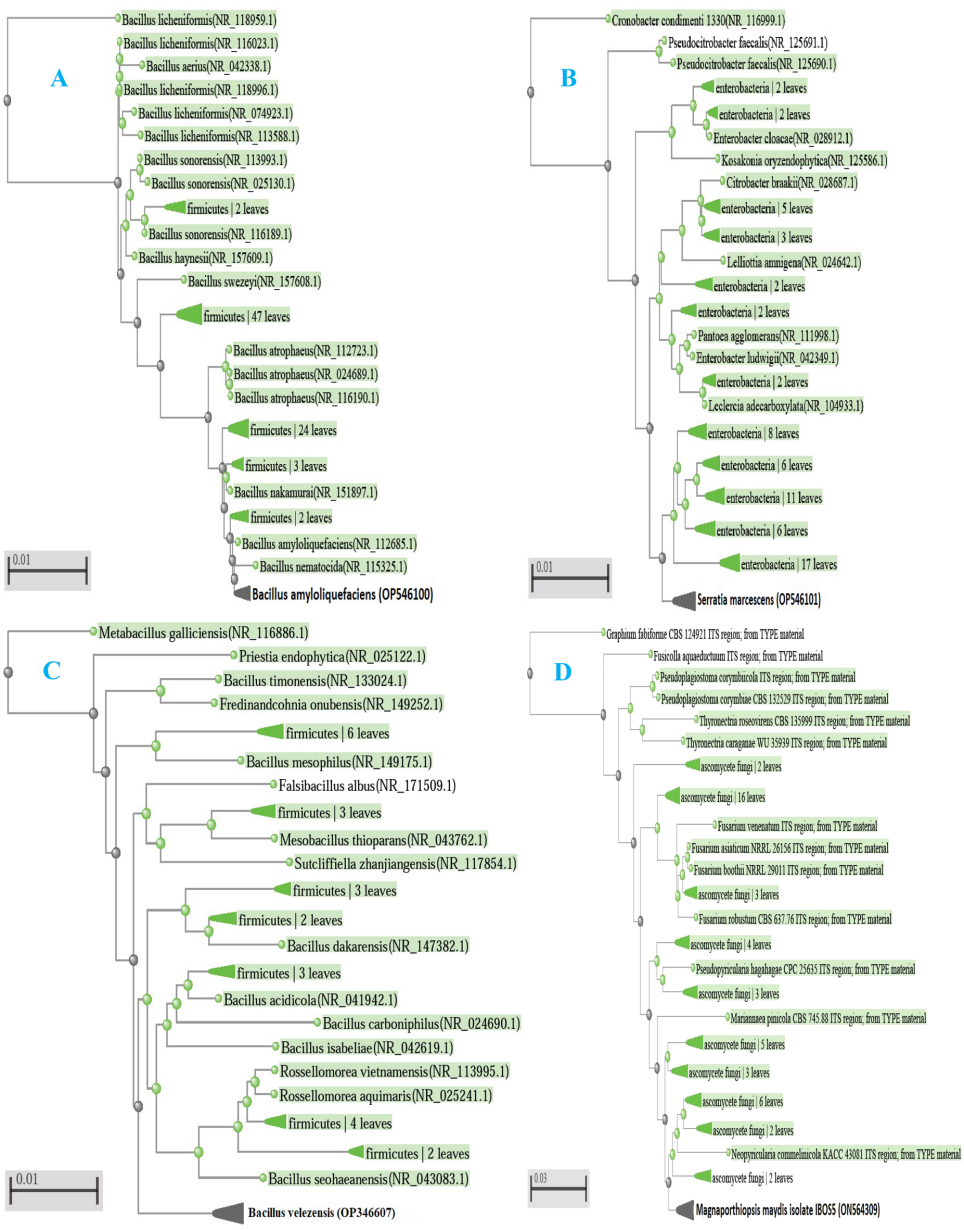
Phylogeny analysis of vermtea bacteria and maize late-wilt pathogen *Magnaporthiopsis maydis*. (**A**–**C**) Phylogenetic tree based on 16S rDNA ITS sequence of *B. amyloliquefaciens*,* Serratia marcescens* and* Bacillus velezensis* (bold) and their closely related species and outgroup retrieved from the literature; (**D**) Phylogenetic tree based on 18S rDNA ITS sequence of *M. maydis* (bold) and its closely related species and outgroup retrieved from the literature.

In vitro tests were performed on 17 bacterial strains obtained from vermitea and tested for their antagonistic activity against *M. phaseolina* and *M. maydis* (Table 1). Among them, three bacterial strains were found to have a significant antagonistic effect against the tested phytopathogens (Fig. 1). *Macrophomina phaseolina* growth inhibition percentages of 54.2, 65.2, and 57.1% were found in PDA plates treated with the three bacterial strains *i.e*., VCB2, VCB7, and VCB11, respectively. These strains had, also, the most antagonistic effect on *M. maydis*, inhibiting growth by 61.7, 74.0, and 87.0%, respectively. Of all, the strains VCB2, VCB7 and VCB11 had the greatest antagonistic effect on both pathogens, showing statistically significant differences respect to the other strains (*P* = 0.05) (Table 1). In contrast to *M. phaseolina*, *M. maydis* was more susceptible to all bacterial strains. The three strains VCB2, VCB7, and VCB11 were discovered to be the most noticeable and effective in inhibiting the growth of both pathogens in plate assays. Sequencing was performed on the PCR products from these strains (VCB2, VCB7, and VCB11), and the sequencing results were then compared for homology with the reported sequences listed in GenBank. These strains showed 99% homology with *Bacillus amyloliquefaciens*, *Serratia marcescens*, and *B. velezensis*, respectively, according to 16S rDNA gene sequence analysis. As a result, these strains were given the accession numbers OP546100, OP546101, and OP346607, respectively, when they added to the GenBank database (Fig. 2A–C).

**Table 1:** Antagonistic activity of bacterial strains from vermitea against *M. phaseolina* and* M. maydis *in vitro after incubation at 27 ± 2 °C for 7 days.

Bacterial strain	*M. phaseolina*		*M. maydis*	
	Linear growth (cm)	Inhibition (%)	Linear growth (cm)	Inhibition (%)
VCB-1	7.74 ± 0.09 c	14.0	6.66 ± 0.16 b	26.0
VCB-2	4.12 ± 0.06 h	54.2	3.45 ± 0.06 g	61.7
VCB-3	6.88 ± 0.05 e	23.5	5.70 ± 0.10 cd	36.7
VCB-4	8.60 ± 0.05 b	04.4	5.76 ± 0.08 cd	36.0
VCB-5	7.73 ± 0.13 c	14.1	5.70 ± 0.05 cd	36.7
VCB-6	8.85 ± 0.03 ab	01.6	5.83 ± 0.06 cd	35.2
VCB-7	3.13 ± 0.00 i	65.2	2.34 ± 0.06 h	74.0
VCB-8	7.71 ± 0.03 c	14.3	6.03 ± 0.23 c	33.0
VCB-9	8.60 ± 0.10 b	04.4	6.83 ± 0.03 b	24.1
VCB-10	7.96 ± 0.03 c	11.5	6.80 ± 0.15 b	24.4
VCB-11	3.86 ± 0.00 h	57.1	1.17 ± 0.12 i	87.0
VCB-12	5.78 ± 0.06 g	35.8	4.86 ± 0.03 f.	46.0
VCB-13	6.53 ± 0.03 f.	27.4	5.73 ± 0.08 cd	36.3
VCB-14	7.75 ± 0.05 c	13.9	5.20 ± 0.05 ef	42.2
VCB-15	7.30 ± 0.30 d	18.9	4.96 ± 0.03 f.	44.9
VCB-16	7.66 ± 0.33 c	14.9	5.46 ± 0.23 de	39.3
VCB-17	7.86 ± 0.06 c	12.7	5.90 ± 0.05 c	34.4
Control	9.00 ± 0.00 a	–	9.00 ± 0.00 a	–

Values are mean of four replicates for each treatment as well as the control. Means ± standard errors within a column followed by the different letters express statistically significant differences (*P* = 0.05) among treatments according to Duncan’s multiple range test**.** The percentage of growth inhibition was calculated using the formula: [(linear growth of the pathogen colony in control dish − linear growth of the pathogen colony in treated dish)/linear growth of the pathogen colony in control dish] × 100.

#### Antifungal activity of wood vinegar

**Figure 3:**
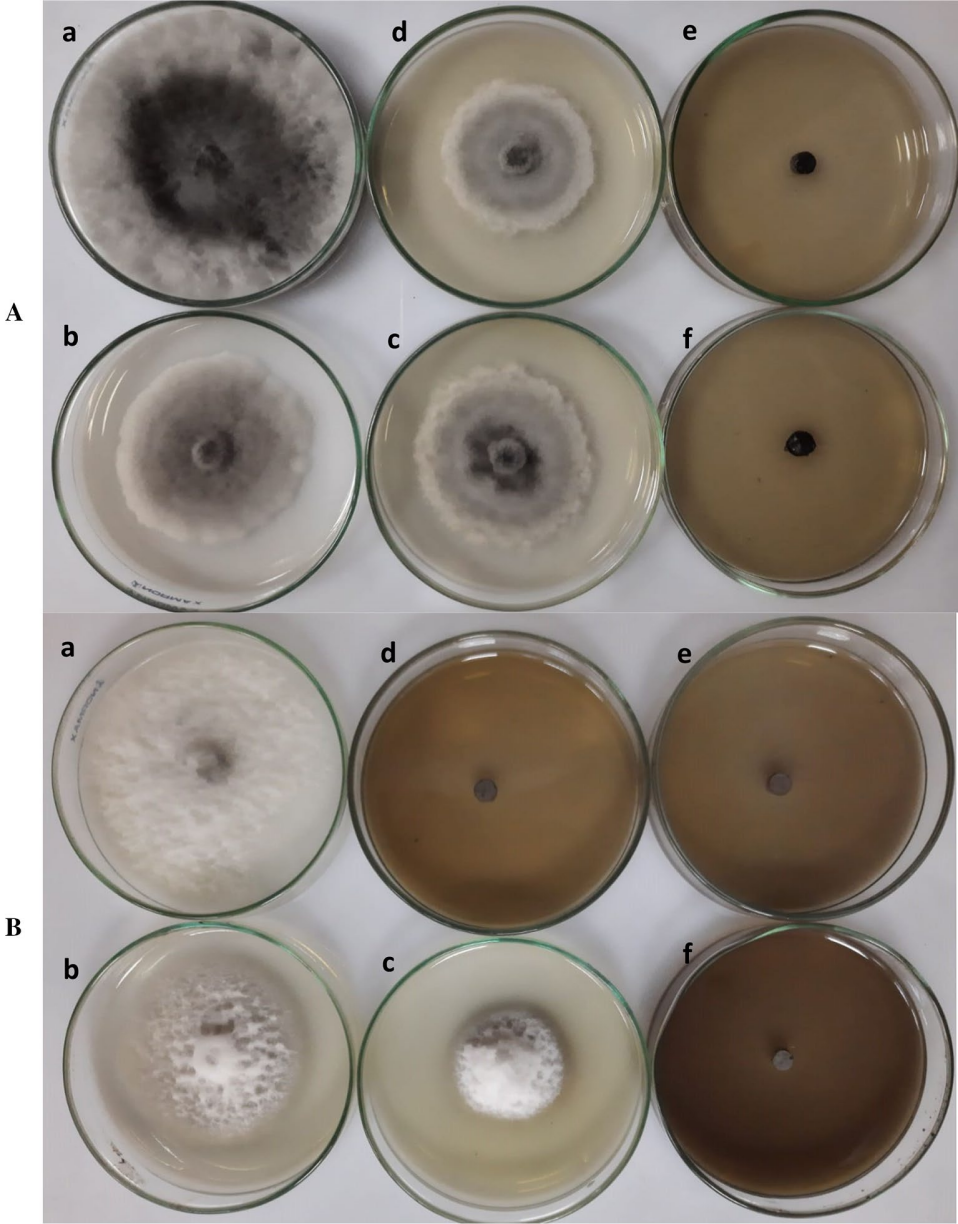
Efficacy of poisoned medium (PDA mixed with wood vinegar) on inhibiting *M. phaseolina* (**A**) and *M. maydis* (**B**) under in vitro conditions at 7 days after inoculation. (**a**) control, (**b**) 0.5% wood vinegar, (**c**) 1.0% wood vinegar, (**d**) 1.5% wood vinegar, (**e**) 2.0% wood vinegar and (**f**) 2.5% wood vinegar.

Table 2 displays the mycelium growth and percentage growth inhibition of wood vinegar against the charcoal-rot fungus *M. phaseolina* and late-wilt fungus *M. maydis* at concentrations ranging from 0.5 to 2.5% (v/v). In both fungi, wood vinegar performed better with increasing concentration and had antifungal activity in all concentrations (Fig. 3). Both pathogenic fungi colonies were able to grow on PDA mixed with wood vinegar at 0.5 and 1.0%. The means of the colony diameters of *M. phaseolina* and *M. maydis* were 7.7, 4.9, and 6.3, 2.8 cm, respectively. On the other hand, *M. phaseolina*, with a colony diameter of 3.8 cm, was able to grow on PDA mixed with wood vinegar at a 1.5%. Notably, hypha grew only upwards and not sideways. At 1.5, 2.0, and 2.5%, wood vinegar inhibited the late-wilt fungus, *M. maydis*, completely. However, at these concentrations, wood vinegar inhibited the charcoal-rot fungus, *M. phaseolina* by 57.8, 100 and 100%, respectively. PDA medium not mixed with wood vinegar showed no inhibition (Fig. 3).

**Table 2:** In vitro antifungal activity of wood vinegar against *M. phaseolina* and* M. maydis* after incubation at 27 ± 2 °C for 7 days.

Wood vinegar (%)	*M. phaseolina*		*M. maydis*	
	Linear growth (cm)	Inhibition (%)	Linear growth (cm)	Inhibition (%)
0.0	9.0 ± 0.00 a	–	9.0 ± 0.00 a	–
0.5	7.7 ± 0.12 b	14.4	4.9 ± 0.13 b	45.6
1.0	6.3 ± 0.14 c	30.0	2.8 ± 0.14 c	68.9
1.5	3.8 ± 0.14 d	57.8	0.0 ± 0.00 d	100
2.0	0.0 ± 0.00 e	100	0.0 ± 0.00 d	100
2.5	0.0 ± 0.00 e	100	0.0 ± 0.00 d	100

Values are mean of four replicates for each treatment as well as the control. Means ± standard errors within a column followed by the different letters express statistically significant differences (*P* = 0.05) among treatments according to Duncan’s multiple range test**.** The percentage of growth inhibition was calculated using the formula: I = ¬(C − T)/C × 100, where I = inhibition, as a percentage; C = colony diameter of mycelium from control Petri-dishes (cm); and T = colony diameter of mycelium from the Petri dishes containing the wood vinegars (cm).

### Greenhouse trials

#### Effect on plant growth promotion

**Figure 4:**
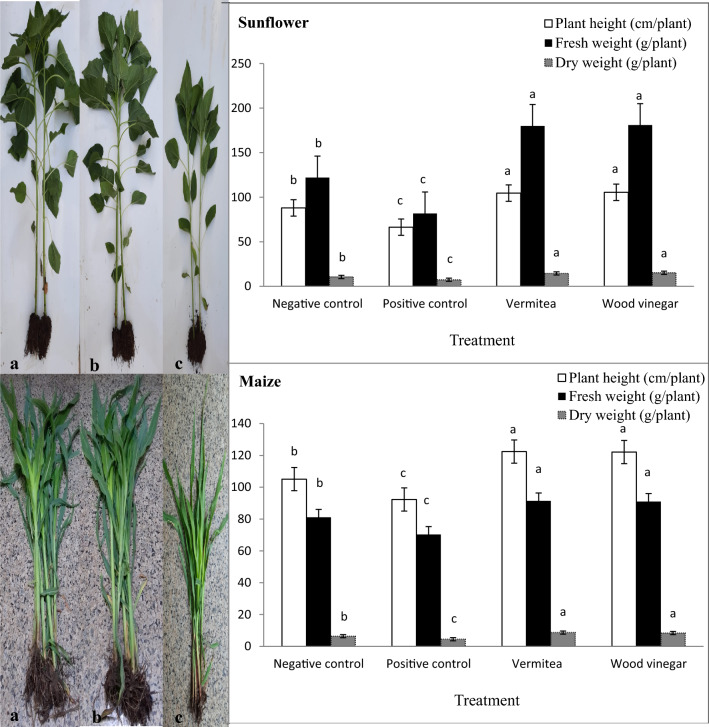
Effect of application with vermitea and wood vinegar on plant growth promotion of sunflower and maize plants, 35 days after sowing, grown under greenhouse conditions. (**a**) vermitea, (**b**) wood vinegar and (**c**) positive control. Values are mean of ten replications for each treatment as well as the positive and negative controls. Bars with the different letters within each variable indicate that the means ± standard errors are significantly different at *P* = 0.05, according to Duncan’s multiple range tests. Before sowing sunflower seeds and maize grains were soaked separately in each of vermitea (1:10, v:v) and wood vinegar (2.0%) for 2 h. After sowing, vermitea (1:10, v:v) and wood vinegar (2.0%) treatments at the rate of 200 mL pot^−1^ were applied four time intervals. The first application was 1 day before sowing. The three remains applications were applied weekly for 3 weeks starting 3 days after sunflower and maize seedlings emergence to the root zone and foliage of plants.

Vermitea and wood vinegar treatments significantly (*P* = 0.05) increased plant height, fresh weight and dry weight of the two crops (sunflower and maize) when compared to controls (Fig. 4). The plant height in the sunflower and maize negative controls was 88.0 and 105.1 cm, respectively, whereas the plant height in the sunflower and maize positive controls was reduced by 24.5 and 12.2% as a result of *M. phaseolina* and/or *M. maydis* inoculation. Treatments with vermitea or wood vinegar significantly increased plant height in comparison to the positive controls in both crops (Fig. 4). Vermitea or wood vinegar treatments against *M. phaseolina* and/or *M. maydis* stress significantly increased plant height in sunflower and maize, which increasing plant height by 36.5; 37.0, and 24.6; 24.4%, respectively. Sunflower and maize plant’s fresh weights were decreased by 32.9 and 13.3%, respectively, after *M. phaseolina* and/or *M. maydis* were introduced to the soil. When compared to the positive control, the vermitea and wood vinegar treatments significantly reduced fungal stress and increased fresh weight of the two crops (Fig. 4). The most significant impact on fresh weight against *M. phaseolina* and/or *M. maydis* stress was provided by these treatments, which had 179.9; 180.9, and 91.4; 91.0 g plant^−1^ in sunflower and maize, respectively (Fig. 4). The negative controls had dry weights of 10.5 and 6.4 g plant^−1^, while the positive controls had dry weights of 7.4 and 4.5 g plant^−1^ of 29.5 and 29.6% reduction, respectively. The vermitea and wood vinegar treatments significantly improved dry weight in sunflower and maize against the two pathogens stress, with 14.5; 15.2 and 8.7; 8.4 g plant^−1^, respectively (Fig. 4).

#### Effect on photosynthetic pigments and antioxidant enzymes

**Figure 5:**
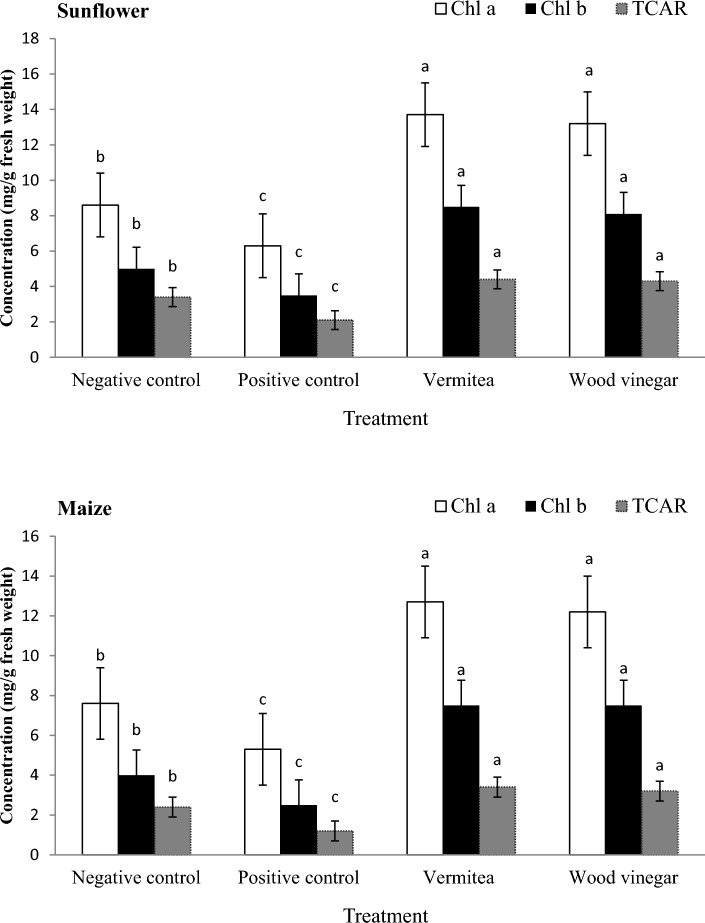
Effect of application with vermitea and wood vinegar on photosynthetic pigments of sunflower and maize plants, 35 days after sowing, grown under greenhouse conditions. Values are mean of ten replications for each treatment as well as the positive and negative controls. Bars with the different letters within each variable indicate that the means ± standard errors are significantly different at *P* = 0.05, according to Duncan’s multiple range tests. Chl a = chlorophyll a, Chl b = chlorophyll a, and TCAR = total carotenoids.

**Figure 6:**
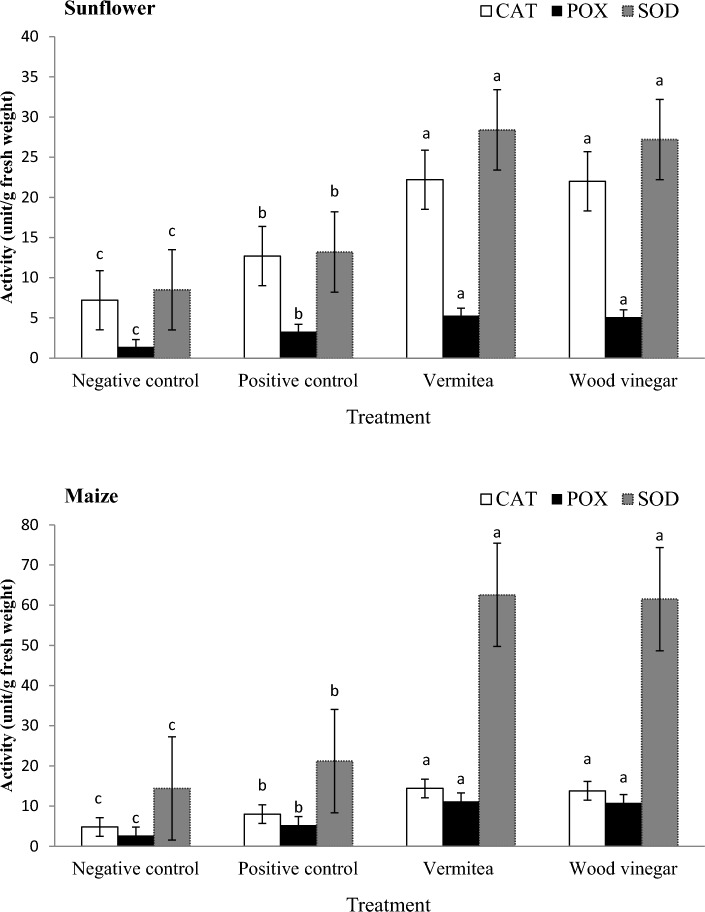
Effect of application with the vermitea and wood vinegar on antioxidant enzymes of sunflower and maize plants, 35 days after sowing, grown under greenhouse conditions. Values are mean of ten replications for each treatment as well as the positive and negative controls. Bars with the different letters within each variable indicate that the means ± standard errors are significantly different at *P* = 0.05, according to Duncan’s multiple range tests. CAT = catalase, POX = peroxidase and SOD = superoxide dismutase.

The effect of vermitea and wood vinegar on the photosynthetic pigments of sunflower and maize plants was studied, and the results were shown in Fig. 5. There were no significant differences in photosynthetic pigment enhancement between vermitea and wood vinegar treatments. *Macrophomina phaseolina* inoculation reduced chlorophyll a, chlorophyll b, and total carotenoids in the positive control by 26.7, 30.0, and 38.2%, respectively, when compared to the negative control of sunflower plants (Fig. 5). When compared to the positive controls, vermitea and wood vinegar increased chlorophyll a, chlorophyll b, and total carotenoids in sunflower plants by 54.0; 52.3, 58.8; 56.8, and 52.3; 51.2%, respectively. Treatments with vermitea and wood vinegar significantly increased the photosynthetic pigment content of maize plants grown in *M. maydis* contaminated soil. When compared to the positive control plants (5.3, 2.5, and 1.2 mg/g), these treatments had the highest chlorophyll a, chlorophyll b, and total carotenoids contents (12.7; 12.2, 7.5; 7.5, and 3.4; 3.2 mg/g) (Fig. 5). The effects of vermitea and wood vinegar on antioxidant enzymes were investigated, with the results shown in Fig. 6. The results reveal differences in enzyme activities between the control and plants grown in *M. phaseolina* or *M. maydis* contaminated soils treated with vermitea or wood vinegar. Plants treated with vermitea and wood vinegar had higher enzyme concentrations than plants grown in *M. phaseolina* or *M. maydis* contaminated soils. Sunflower plants grown in *M. phaseolina*-contaminated soil treated with vermitea or wood vinegar showed 12.2; 12.2, 5.3; 5.1, and 28.4; 27.2 U/g of CAT, POD, and SOD activity, respectively, compared to 12.7, 3.3, and 13.2 U/g in the positive control (Fig. 6). The CAT, POD, and SOD activity of maize plants grown in *M. maydis* contaminated soil treated with vermitea or wood vinegar was 14.4; 13.8, 11.2; 10.8, and 62.6; 61.5 U/g, respectively, compared to 8.0, 5.3, and 21.2 U/g in the positive control.

#### Effect on charcoal- rot and late -wilt incidence

**Figure 7:**
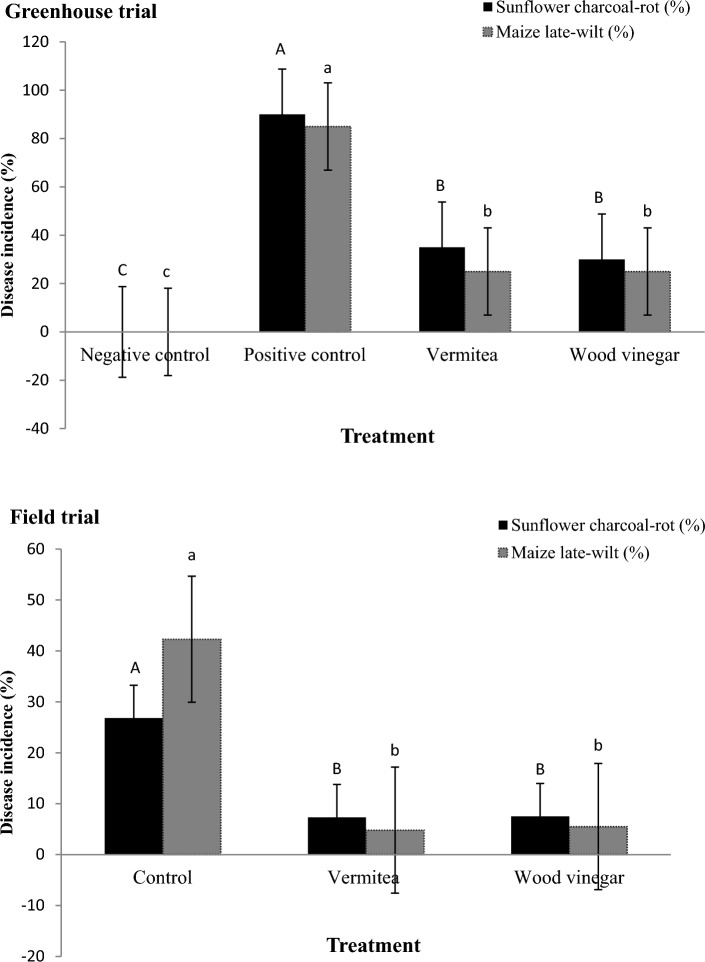
Effect of application with vermitea and wood vinegar on sunflower charcoal-rot and maize-late wilt under greenhouse and field conditions. In greenhouse, values are mean of ten replications for each treatment as well as the positive and negative controls. In field, values are mean of four replications for each treatment as well as the controls. Bars with the different letters within each variable indicate that the means ± standard errors are significantly different at *P* = 0.05, according to Duncan’s multiple range tests. Percentages data of disease incidence were transformed into arcsine square-root transformation for analyses of variance, however untransformed data are presented.

There was no disease found in either the sunflower or maize negative controls. Positive controls, on the other hand, had disease incidence of 90.0 and 85.0% for sunflower charcoal-rot and maize late-wilt, respectively (Fig. 7). Treatments with vermitea or wood vinegar reduced the occurrence of both diseases significantly. In sunflower, vermitea or wood vinegar treatments resulted in disease incidence of 35.0 and 30.0%, respectively. With a 25.0% incidence of late-wilt in maize, vermitea or wood vinegar treatments were found to be effective against *M. maydis* stress (Fig. 7). The wood vinegar and vermitea did not differed significantly.

### Field trials

#### Effect on charcoal-rot and late-wilt incidence

Throughout the crop season, diseased plants with sunflower charcoal-rot and maize late-wilt symptoms were observed in the field experiment. When compared to controls, treatment with vermitea or wood vinegar significantly reduced disease development in both crops (Fig. 7). In contrast, the progression of charcoal-rot and late-wilt diseases was unaffected, and severe disease symptoms appeared 60–80 days after sowing in control plants that had not been treated with vermitea or wood vinegar. As a result, the control set had a high mean disease incidence value of 26.8% for sunflower charcoal-rot and 42.3% for maize late-wilt, respectively (Fig. 7). When compared to the control, all treatments in the case of sunflower significantly decreased the incidence of charcoal-rot. The percentage reduction in charcoal-rot in the vermitea and wood vinegar treatments was 72.8 and 72.0%, respectively. In comparison to control plots of maize, vermitea and wood treatments showed a significant level of disease suppression, with 88.7 and 87.0%, respectively. There was no significant difference in overall disease control efficacy between vermitea and wood vinegar.

#### Effect on plant yield

**Figure 8:**
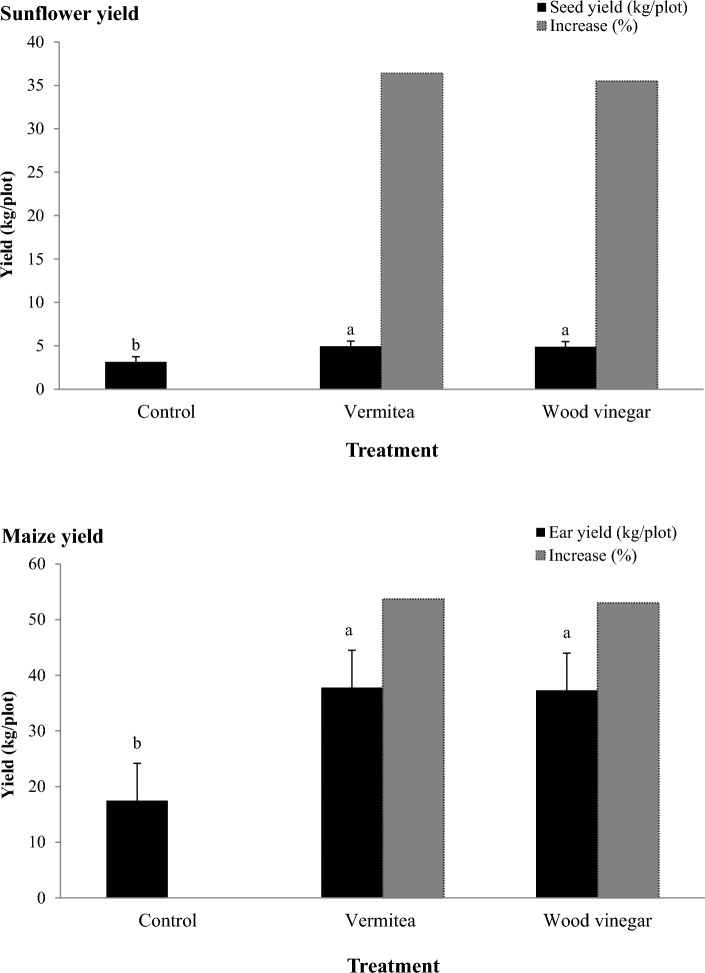
Effect of application with vermitea and wood vinegar on sunflower and maize yield under field conditions. Values are mean of four replications for each treatment as well as the controls. Bars with the different letters within each variable indicate that the means ± standard errors are significantly different at *P* = 0.05, according to Duncan’s multiple range tests.

In general, all treatments significantly increased sunflower and maize yield over the non-treated control (Fig. 8). In sunflower, the seeds yield resulted from the vermitea and wood vinegar treatments was 4.95 and 4.89 kg plot^−1^, respectively, compared to 3.15 kg plot^−1^ in the control. In maize, vermitea (37.8 kg plot^−1^) and wood vinegar (37.3 kg plot^−1^) treatments produced the highest ears yield, which was significantly higher than the non-treated control (17.5 kg plot^−1^) treatment.

## Discussion

In the current study, a total of seventeen bacteria strains from vermitea were tested for their antifungal activity to *M. phaseolina* and *M. maydis*, the pathogens responsible for sunflower charcoal-rot and maize late-wilt, respectively. Surprisingly, these isolates showed promising biological control potential against both pathogens. Three bacterial strains were chosen for molecular characterization due to their high antifungal activity against *M. phaseolina* and *M. maydis*. They were identified as *Bacillus amyloliquefaciens* OP546100, *Serratia marcescens* OP546101, and *Bacillus velezensis* OP346607. Many researchers have discovered that *B. amyloliquefaciens*, *S. marcescens* and *B. velezensis* are effective in plant disease prevention and control^30–32^. Beneficial microbes are in abundance in vermitea, which has a wide variety of bacteria that help with the biocontrol of diseases caused by different soil borne pathogens^33^. For instance, *Fusarium oxysporum* f. sp. *ciceri*, which causes *Fusarium* wilt of chickpeas, was able to be inhibited by 33 actinomycetes strains that were isolated from 25 different herbal vermicomposts^34^. Pathma and Sakthivel^35^ discovered 96 bacterial strains from vermicompost that had antagonistic effect against phytopathogenic fungi. Liu et al.^36^ reported that a total of 28 bacterial strains from vermicompost exhibited antagonistic activity against *Fusarium oxysporum* f. sp. *cucumerinum* (FOC) in cucumber. Soltan et al.^37^ found that the ten bacterial isolates obtained from vermicompost had an in vitro antagonistic effect against *Fusarium solani*, *Fusarium* spp., *M. phasolenia* and *Rhizoctonia solani*.

The wood vinegar as biochar derivatives used in this study performed best at 1.5 and 2.0%, which completely inhibiting the both pathogens. Wood vinegar has the ability to inhibit pathogenic fungi due to its chemical properties, including acidity and other chemical components. It has been demonstrated that the components of wood vinegar like acetic acid, formaldehyde, and methanol kill fungi, bacteria, and virus particles^38,39^. In the present study, the highest antifungal activity of wood vinegar was found to be associated with total phenol, total organic acids and total alcohols (Table 3). In comparison to the charcoal-rot fungus *M. phaseolina*, the antifungal activity of wood vinegar was significantly higher against late-wilt fungus *M. maydis*. Even at the highest dosage (1.5%) of wood vinegar, *M. phaseolina* only slightly expanded (57.8% inhibition). Chalermsan and Peerapan^40^ found that some pathogenic fungi, such as *R. solani*, *Sclerotium oryzae*, *Helminthosporium maydis*, *Pythium* sp., *Colletotrichum gloeosporioides*, and *Choanephora cucurbitarum*, were inhibited by wood vinegar solutions on PDA medium. Matnork et al.^29^ discovered that wood vinegar at 1.0% completely inhibited the mycelial growth and sclerotial germination of *S. rolfsii*.

**Table 3:** The important physical and chemical parameters in the potted soil, vermicompost and wood vinegar used in the present study.

Soil		Vermicompost		Wood vinegar	
Parameter	Content	Parameter	Content	Parameter	Content
pH	7.32	pH	8.57	pH	2.99
EC (dSm-1) (1:10)	1.29	EC (dSm^−1^) (1:10)	1.32	Acetic acid (µg/ml)	4.12
mEq /L Ca^++^	3.80	Organic carbon (%)	29.25	formic acid (µg/ml)	3.16
mEq /L Mg^++^	2.33	Organic matter (%)	47.56	Butyric acid (µg/ml)	3.18
mEq /L Na^+^	2.44	C/N ratio	15.96:1	Propionic acid (µg/ml)	12.35
mEq /L K^+^	0.36	Total N (%)	1.77	Phenol (µg/ml)	5.37
mEq /L HCO^−^_3_	1.7	Total P (%)	1.22	Nitrogen (%)	0.08
mEq /L Cl^−^	2.30	Total K (%)	1.17	Potassium (ppm)	1739.0
mEq /L SO_4_	3.86	Iron (ppm)	0.25	Phosphorus (ppm)	26.7
Available N (ppm)	137.2	Manganese (ppm)	0.42	Iron (ppm)	1.65
Available P (ppm)	23.6	Zinc (ppm)	0.43	Manganese (ppm)	42.6
Available K (ppm)	312.5	Ash (%)	45.67	Zinc (ppm)	2.78
Iron (ppm)	4.5	Moisture (%)	70.56	Weeds	0.0
Manganese (ppm)	7.0	Weeds	0.0	Nematodes	0.0
Zinc (ppm)	1.4	Nematodes	0.0	Pathogenic fungi	0.0
Clay (%)	49.36	Pathogenic fungi	0.0	–	–
Silt (%)	43.56	–	–	–	–
Sand (%)	7.08	–	–	–	–

In this study, the application of vermitea or wood vinegar reduced the incidence of sunflower charcoal-rot and maize late-wilt diseases in both greenhouse and field trials. This study suggests that indigenous vermitea bacteria and wood vinegar inhibitor components may be involved in this suppression. From vermitea, we isolated 17 bacterial strains. *B. amyloliquefaciens*, *S. marcescens*, and *B. velezensis* were identified and having high antagonistic activity against the causal pathogens. These bacterial strains exert biocontrol effects through a variety of mechanisms, including direct antibiosis and competition through the secretion of a variety of secondary metabolites in the rhizosphere, as well as stimulation of plant-induced systemic resistance^41^. The results are in line with a number of recent studies. Sabaté et al.^42^ discovered that in the presence of *M. phaseolina*, *B. amyloliquefaciens* B14 synthesized various lipopeptides such as surfactin, iturin, fengycin, kurstatin, and polymyxin, implying that they are the primary cause of the antagonistic effect observed. Chen et al.^43^ reported that *B. velezensis* ZW-10 liquid culture and cell-free culture filtrate had significant inhibitory effects on rice blast. In Korea, Kim et al.^44^ reported that *B. velezensis* has biocontrol activity against apple bitter rot. Also, *B amyloliquefaciens* AW3 causes mycelial cell malformation, swelling, and distortion in the fungus *Fusarium oxysporum*^45^. According to the obtained data, the fungicidal activity of wood vinegar against the occurrence of charcoal-rot and late-wilt might be caused by a combination of its acidic nature and the presence of components like phenolic, polyphenolic compounds, and organic acids. Wood vinegar has the capacity to inhibit the two pathogenic fungi because of its chemical components such as acetic acid, formaldehyde and methanol, as previously reported^40^. Using GC–MS analysis, Velmurugan et al.^46^ identified seven major compounds in neutralized wood vinegar and eleven major compounds in acidic wood vinegar. Moreover, Mahmud et al.^47^ reported that the presence of phenols and their major derivatives, as indicated by GC–MS and FTIR analysis, is what gives pyroligneous acid its antifungal properties.

It has been reported that the induction of antioxidant enzymes is associated with plant tolerance to fungal disease stress. Thus, in *M. phaseolina* or *M. maydis* infested soil, the antioxidant activity of vermitea and wood vinegar treated sunflower and maize was investigated. The activity of CAT, POD, and SOD enzymes were differed from the negative control, positive control, and plants grown in pathogen-contaminated soils treated with vermitea or wood vinegar. Plants treated with vermitea or wood vinegar had higher concentrations of enzyme activities than both the negative and positive controls. According to Mahmud et al.^47^, wood vinegar may have a second mode of action in disease control in addition to direct antifungal activity, which could be the induction of resistance in plants to particular diseases. Furthermore, Rathika et al.^48^ found that the vermi-wash treatment increased the antioxidant enzyme activity in *Sorghum bicolor* plants (140, 125, and 152 U/mg of CAT, SOD, and POD, respectively). Plant polyphenols act as reducing agents, scavengers, metal chelators, and oxygen radical quenchers in the cell to provide antioxidant activity. Antioxidant enzymes catalyze the reaction that involves the breakdown of H_2_O_2_, in which one molecule of H_2_O_2_ acts as a substrate donor and another molecule of H_2_O_2_ acts as an oxidant or electron acceptor. This molecule may be in charge of producing stress defense signals because an increase in catalase causes an increase in H_2_O_2_. In addition, H_2_O_2_ and other free radicals are toxic to a wide range of microbial pathogens^49^. When a plant interacts with a pathogen, the oxidative potential of H_2_O_2_ helps to produce lignin through the peroxidase-mediated crosslinking of structural proteins rich in proline and phytoalexin biosynthesis as well as the polyphenoloxidase-mediated conversion of O-dihydroxyphenols to harmful O-quinones^50^.

In the current study, the application of vermitea and wood vinegar improved the growth and photosynthetic pigments of sunflower and maize plants grown in pots trials. In addition, these treatments increased the yield of both crops in field trials. This is because vermitea and wood vinegar had beneficial properties. Ruiz et al.^51^ discovered that inoculating vermicompost tea with plant growth promoting bacteria resulted in significant increases in tomato fruit yield and quality. Mineral nutrients in plant-available forms, a hormone-like effect on plant growth, and stimulation of plant mineral nutrition are all potential beneficial mechanisms of vermitea and wood vinegar on plants^47,52^. Increased leaf photosynthetic pigment content (chlorophyll and carotenoid) of sunflower and maize as a result of vermitea treatment can be used to indicate improved plant physiological state^53,54^. This effect was also observed in the wood vinegar treatment in the current study as well as in other experiments^55,56^, indicating that vermitea and wood vinegar components activate photosynthesis-related processes. When used as a foliar fertilizer, wood vinegar increases cucumber, lettuce, and cole yields^55,56^. Tikoria et al.^57^ reported that when tomato seedlings were treated with 100% vermicompost extract, root and shoot length, root and shoot fresh weights increased by 28, 36.53, 68.20 and 43%, respectively.

## Conclusion

The most harmful pathogens that severely reduce sunflower and maize plant yields are *M. phaseolina* and *M. maydis*, respectively. The effectiveness of vermitea and wood vinegar in preventing these fungi-related diseases was assessed in this study. Out of 17 bacterial strains obtained from vermitea, three demonstrated the strongest inhibitory effect against *M. phaseolina* and *M. maydis*. Obtained data also stated that wood vinegar, as biochar derivative, prevented the representative pathogens from growing in in vitro tests. The greenhouse and field results showed that vermitea and wood vinegar were effective in preventing sunflower charcoal-rot and maize late-wilt, while also significantly increased plant growth, antioxidant enzymes, photosynthetic pigments and yield for both crops when compared to the untreated control. This discovery raises the possibility that vermitea and wood vinegar may work in two different ways to combat charcoal-rot of sunflower and late-wilt of maize. Direct antifungal activity and the development of plant resistance to the responsible pathogens are two examples of these. To increase the use of vermitea and wood vinegar in agricultural production, more thorough studies are required.

## Materials and methods

### Soil, vermicompost and wood vinegar

The greenhouse experiment took place in clay soil located at El-Gharbia governorate, Egypt. The vermicompost used in this study was provided by the Environment and Bio-Agricultural Department, Faculty of Agriculture, Al-Azhar University, Cairo, Egypt. It consisted of horse dung and mushroom compost 1:1 (v:v) processed in outdoor beds by earthworms *Eisenia foetida*. The study's wood vinegar was made from *Psidium guajava* wood and obtained from the Egyptian Company for Modern Agriculture in Cairo, Egypt. The best pyrolysis conditions were attained by heating at a rate of 1.4 °C per minute to a final temperature of 550 °C. Wood vinegar was infused through Whatman filter paper No. 1 and sterilized using a 0.22 μm filters syringe before being tested for antifungal activity. The soil, vermicompost, and wood vinegar were chemically analyzed using standard procedures at the Soils, Water & Environment Research Institute, Agriculture Research Centre, Giza, Egypt. Table 3 shows the chemical properties of soil, vermicompost and wood vinegar used in this study.

### Sunflower charcoal-rot pathogen

**Figure 9:**
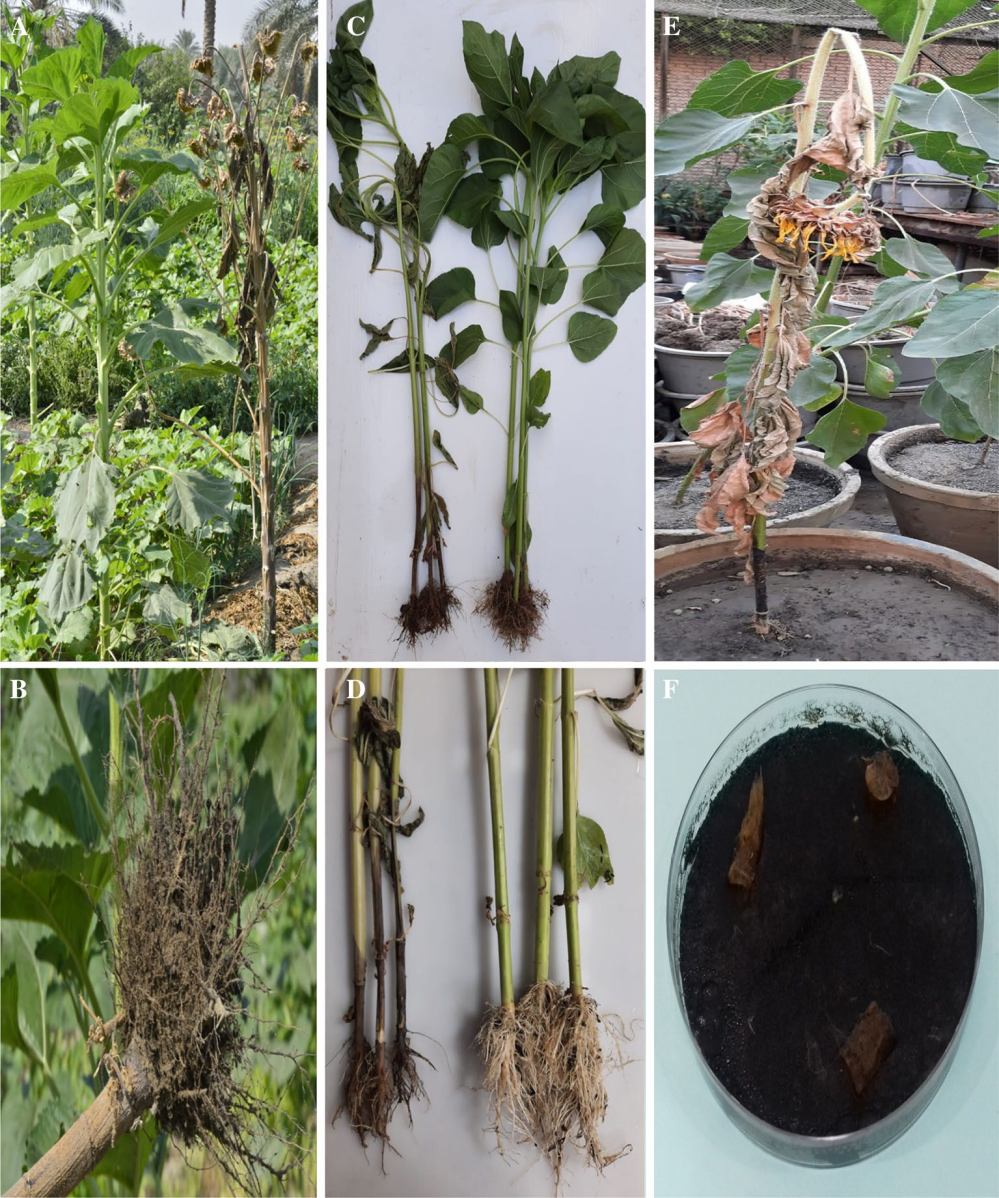
Typical natural symptoms of charcoal-rot on sunflower plant behind healthy one (**A**). The black microsclerotia are visible on root and the lower part of stem of diseased plant (**B**). Symptoms of charcoal rot disease on foliage and root parts of sunflower plants artificially infected with* M. phaseolina* isolate, 35 days after sowing (**C**) (left: infected plants and right: healthy plants). Charcoal-rot disease signs on infected stem and roots (left: infected plants and right: healthy plants) (**D**). Symptoms and signs of charcoal-rot disease on foliage and root parts of sunflower plants artificially infected with* M. phaseolina* isolate, 68 days after sowing (**E**) Pure culture of *M. phaseolina* isolate grew on the PDA medium (**F**).

In the second half of August and later in 2021, diseased samples exhibiting symptoms of charcoal-rot of sunflower (cv. Sakha 53) were collected from some infected field localities in El-Gharbia Governorate, Egypt. Infected sunflower plants died early and took on a silvery gray appearance (Fig. 9A). In the lower portion of their stem, black microsclerotia were found (Fig. 9B). The infected stem and root samples were washed by running tap water and then cut into small (1 cm) pieces. By dipping the pieces in 0.5% sodium hypochlorite for 45 s, followed by 3 min of washing with sterile distilled water, the pieces were surface sterilized. After drying on sterilized filter paper, the fragments were kept on 90 mm Petri-plates of sterilized potato dextrose agar medium (PDA, Difco, Detroit, MI, USA) that has been modified by adding chloramphenicol as antibacterial agent then incubated at 25 ± 2 °C for 7 days. The fungus was identified based on host plant symptoms, colony color, and the morphology of the microsclerotia and pycnidia after the colonies were purified using single hyphal tip techniques. Sunflower seeds were planted in 35-cm diameter pots (2 seeds/pot) with sterilized soil that had been infused with the previously obtained strain of *M. phaseolina* under greenhouse conditions. For a period of 12 weeks, sunflower plants were watched for indications of charcoal-rot. Ninety days after sowing, 95.0% of the *M. phaseolina*-inoculated pots had disease incidence (dead plants as a result of charcoal-rot). Sclerotia in the plant base, a symptom of charcoal-rot, are present on all infected plants (Fig. 9E). The negative control (non-infested pots) did not induce disease (Fig. 9C and D). In order to confirm Koch^’^s hypotheses, *M. phaseolina* was once again isolated from infected tissues of cultivated sunflower plants. The infected stem inoculated on PDA produced grey hyphae that eventually turned black and formed microsclerotia (Fig. 9F).

### Maize late-wilt pathogen

A virulent isolate of *Magnaporthiopsis maydis* was used. Isolation, morphology identification and pathogenicity test of this isolate was confirmed in previous study^8^. In this study, the molecular identification of this isolate was verified. Whole genomic DNA was extracted using the i-genomic BYF DNA extraction Mini Kit as directed by the manufacturer (iNtRON Biotechnology Inc., South Korea). PCR amplification and sequencing of transcribed spacers (ITS), using the forward primer ITS1 (5′ TCCGTAGGTGAACCTGCGG-3′) and the reverse primer ITS4 (5′ TCCTCCGCT TATTGATATGC-3′), were used to identify the pathogen molecularly^58^. A polymerase chain reaction (PCR) was used to amplify a particular region of the rDNA primer (2 min at 94 °C, followed by 35 cycles of denaturation at 95 °C for 30 s, annealing at 56 °C for 30 s, elongation at 72 °C for 60 s, and final extension at 72 °C for 10 min). The amplified products were separated on 1% agarose gels in 1X TBE (Tris–borate-EDTA) buffer for approximately 2 h at a constant voltage of 100 V. The nucleotide sequence similarity of the PCR products was examined using BLAST (http://www.ncbi.nlm.nih.gov) analysis against the National Center for Biotechnology Information database after the PCR products were sent to GATC Company's sequencing laboratories in Germany for analysis. The phylogenetic trees showed that this isolate shared many characteristics with the isolate of the *M. maydis* type that was deposited in the NCBI Center and uploaded to GenBank under the accession number ON564309 (Fig. 2D).

### Laboratory experiments

#### Isolation and antagonistic activity of vermitea bacteria

In this study, the vermitea was prepared from vermicompost (cured for 3 months). For vermitea extraction procedures, 1 L of vermicompost was mixed with 10 L of deionized water (25–30 °C). The vessel was a typical polymer container with an air pump and interior tubings that enabled thorough mixing and simultaneous aeration of the liquid inside, but no thermostating. The extraction time was 24 h unless otherwise stated. To boost microbial growth, 1 g L^−1^ of dry humic acid and 0.5 g L^−1^ of citric acid were added before extraction. Just before application, the vermitea was filtered through a nylon membrane. According to Mu et al.^22^ and Darwesh et al.^59^, total culturable bacteria from vermitea sample were counted by dilution plating onto Nutrient Agar (NA) medium and purified on Luria–Bertani (LB) agar medium. The purified bacterial strains were examined for antagonistic activity against *M. phaseolina* and *M. maydis* using the dual culture method^60^. The bacterial strains were streaked on one side of a Petri dish (1 cm from the edge) containing PDA medium, and a 5 mm mycelial disc from a 7-day old pathogenic fungal culture was placed on the opposite side. The plates were incubated for 7 days at 27 ± 2 °C. Only dishes inoculated with the pathogen were used as control. After an incubation period, the linear growth of the pathogen was assessed. Each treatment was replicated four times over the course of the experiment. According to the following formula the percentage of growth inhibition was calculated as follow: Growth inhibition % = (Linear growth of the pathogen colony in the control- linear growth of the pathogen colony in the treatment/ Linear growth of the pathogen colony in the control) × 100.

#### Molecular identification of vermitea bacteria

The strains showing the greatest inhibition in in vitro tests were chosen. The morphology and biochemical tests on the bacterial strains were carried out utilizing Bergey's Manual of Systematic Bacteriology ^61^. For molecular identification, lysozyme (20 mg/mL) and proteinase K (1 mg/mL) were used to extract genomic DNA ^33^. Total genomic DNA was purified using isopropanol buffer. The 16S rRNA genes were amplified using extracted DNA and 2 primers, F: (5'- AGAGTTTGATCCTGGCTCAG -3') and R: (5'- TACGGTTACCTTGTTACGACTT -3'). Initial denaturation at 95°C for 5 min was followed by 35 cycles of 95°C for 30 s, 55°C for 30 s, and 72°C for 45 s to complete extension in the PCR amplification. The QIAquick Gel Extraction Kit (QIAGEN, USA) was used to purify the PCR product before it was run on an agarose gel for sequencing. The BLAST program was used to identify bacterial strains (National Centre for Biotechnology Information). The Jukes Cantor Model was used to align the sequences. The neighbor joining (NJ) algorithm was used for the phylogenetic reconstruction, along with bootstrap values, and the results were submitted to Gene Bank.

#### Antifungal activity of wood vinegar

The fungal inhibition bioassay of wood vinegar against *M. phaseolina* and *M. maydis* was performed according to previous manuscript ^39^. *Macrophomina phaseolina*, a charcoal-rot fungus, and *M. maydis*, a late-wilt fungus, were grown on PDA medium in dishes for 7 days at 25 ± 2 °C and used as inoculants. PDA medium was autoclaved at 121 °C for 15 min. It was then amended with 0.5, 1.0, 1.5, 2.0, and 2.5% (v/v) wood vinegar before being poured into Petri-dishes. The Petri-dishes were then cooled before being centrally inoculated with a single plug (5 mm in diameter) from the pre-cultured PDA plate of each fungus. As controls, PDA dishes containing zero percent wood vinegar were used. Each fungus was replicated four times, once for the control and once for each concentration. Dishes with the treated and untreated ingredient were incubated at 27 ± 2 °C for 7 days. The percentages of mycelial growth inhibition were assessed as mentioned before.

### Greenhouse experiments

Pot experiments were carried out in an open greenhouse during the summer of 2022 at the Plant Pathology Department of the National Research Centre in Giza, Egypt. The experiment's minimum and maximum temperatures were 20 to 30 °C and 32 to 40 °C, respectively. We bought maize grains cv. Landraces and sunflower seeds cv. Sakha 53 from the Egyptian Agricultural Research Center, Egypt.

#### Preparation of pathogens inocula

Inoculants were grown on potato dextrose agar (PDA) and potato dextrose agar yeast extract (PDAY) dishes for 7 days at 25 ± 2 °C for *M. phaseolina*, a charcoal-rot fungus, and *M. maydis*, a late-wilt fungus. A 1000 mL Erlenmeyer flask containing 200 g of wet, sterile sorghum grain was inoculated with 10 colony agar disks (5 mm in diameter each), taken from the edges of a 7-day-old culture, to create inocula for each fungus. Cultures were incubated in the incubator for 4 weeks in a dark at 25 ± 2 °C. The inoculum was then collected and homogenized.

#### Preparation of pots

The experiment was conducted in 35 cm diameter pottery pots filled with 8 kg sterilized clay soil that had been pre-mixed with 4% inocula of any of *M. phaseolina* and *M. maydis*. Negative control treatment pots were made of non-infested soil. The soil and pots were sterilized before filling with a formalin solution made up of 2.5 L of concentrated solution (40% formaldehyde) to 50 L of water. The soil was then covered with a plastic sheet for 7 days to keep the gas in place. The soil was not planted until the formaldehyde odor had subsided. After filling, the pots were watered, and a week was given for the inoculum to establish. Sunflower seeds and maize grains were sown in experimental units (pots) on May 20, 2022, at a rate of 5 seeds or grains per pot. Twelve days later, plants of each crop were thinned to two plants per pot. Each pot for each crop received 15 g of phosphate rock (P_2_O_5_) and 8.0 g of ammonium nitrate prior to sowing. Ammonium nitrate fertilizer was applied at rates of 8.0 g pot^−1^ at 20, 30, and 40 days after sowing, and plants were irrigated when it was necessary.

#### Treatments and experimental design

Before planting, sunflower seeds and maize grains were soaked separately for 2 h in vermitea (1:10, v:v) or wood vinegar (2.0%). Four times after sowing, vermitea (1:10, v:v) or wood vinegar (2.0%) treatments were applied at a rate of 200 mL pot^−1^. The first application was occurred out 1 day prior to sowing. The three residue applications were applied weekly for 3 weeks, beginning 3 days after the emergence of sunflower and maize seedlings to the root zone and foliage of plants. Half of the vermitea and/or wood vinegar were applied to the root zone and the other half to the foliage; however, once the foliage was saturated, the solutions applied to the foliage were moved to the root zone. In the greenhouse experiments, a randomized complete block design (RCBD) with 10 replications was used. Vermitea application, wood vinegar application, negative control (non-treated seeds or grains sown in uninfested and untreated soil), and positive control were the treatments used for each crop. For every crop, there are two separate experiments. The first experiment examined the effects of vermitea and wood vinegar on plant physiological traits and growth promotion 35 days after planting. In order to determine the impact of vermitea and wood vinegar on the incidence of charcoal-rot and late-wilt, a second experiment was conducted 90 days after planting.

#### Experiment I: effect on physiological traits and growth promotion

To investigate the effect of vermitea and wood vinegar applications on physiological traits (photosynthetic pigments and antioxidant enzyme activities) and growth promotion of sunflower and maize, the following four treatments were set up, each with 10 replications for each crop:

**Table:** 

	Sunflower	Maize
T1,	Negative control	Negative control
T2,	Positive control (*M. phaseolina*)	Positive control (*M. maydis*)
T3,	Vermitea application	Vermitea application
T4,	Wood vinegar application	Wood vinegar application

The sunflower and maize plants were pull-off 35 days after they were planted by mulching them out of their pots. Plant height (cm plant^−1^) and fresh weight (g plant^−1^) as well as dry weight (g plant^−1^) were measured for each crop. Before mulching, sunflower and maize leaf samples were separated for the analysis of photosynthetic pigments and antioxidant enzyme activity. According to Porra^62^, photosynthetic pigments [Chlorophyll: (Chl a, Chl b, and total carotenoids (TCAR)] were measured in fresh sunflower and maize leaves and the results were expressed as mg/g^−1^ fresh weight. The extraction and determination of antioxidant enzymes were carried out^63^, and the activity was expressed as unit/g fresh weight/min. At 240 nm, catalase activity (EC 1.11.1.6) was detected. By monitoring the rise in absorbance at 470 nm brought on by guaiacol oxidation, the activity of peroxidase (EC 1.11.1.7) (POX) was ascertained. At 560 nm, superoxide dismutase (EC 1.15.1.1) activity (SOD) was measured using the photochemical technique.

#### Experiment II: effect on charcoal-rot and late-wilt incidence

To determine the impact of vermitea and wood vinegar applications on the incidence of sunflower charcoal-rot and maize late-wilt diseases, the previous treatments were set up with ten replications for each. At the end of the experiment (90 days after planting), the percentage of infected plants during the growing season was calculated as follows: Charcoal-rot and/or late-wilt infection (%) = (number of dead plants due to the disease during the growing season/total number of plants) × 100.

### Field experiments

The goal of the experiment was to ascertain how vermitea and wood vinegar affected both the incidence of diseases like sunflower charcoal-rot and maize late-wilt as well as the yield of both crops. The trial took place in the Egyptian Governorate of El-Gharbia from May to August 2022. The experiment's natural soil type was clay. The contiguous maize-growing region that made up this field site stood out because it was naturally infested with a high *M. maydis* inoculum and suffered from severe late-wilt disease^8^. Before seeding, soil samples were taken, and the Mihail and Alcorn^64^ method was used to check for the presence of *M. phaseolina* isolates. A fungus population was discovered in the field. The experiment was set up in a randomized block design for each crop, with three treatments and four replications (plots). Sunflower seeds cv. Sakha 53 and maize grains cv. Landraces were sown with hand drill in holes (two seeds/hole) and a plot size of 4 lines each equaling 0.75 m width × 5.0 m length was set up. Each crop's seedlings were thinned 7 days after emergence to maintain a plant-to-plant distance of 20 cm and a row-to-row distance of 75 cm. As per traditional sunflower and maize cultivation technology, recommended agronomic practices such as hoeing, weeding, inorganic fertilizer (N:P:K) doses, and insect management were carried out as and when required. The following were the applications of vermitea and wood vinegar: Before planting, sunflower seeds and maize grains were soaked separately for 2 h in vermitea (1:10, v:v) or wood vinegar (2.0%). Four times after sowing, vermitea (1:10, v:v) and wood vinegar (2.0%) treatments were applied at a rate of 100 mL hole^−1^. The first application occurred immediately following sowing and before irrigation. The three residue applications were applied weekly for 3 weeks, beginning 3 days after the emergence of sunflower and maize seedlings to the root zone and foliage of plants. Half of the vermitea and/or wood vinegar were applied to the root zone and the other half to the foliage; however, once the foliage was saturated, the solutions applied to the foliage were moved to the root zone. The following treatments were used on each crop:

**Table:** 

	Sunflower	Maize
T1,	Un-treated control	Un-treated control
T2,	Vermitea application	Vermitea application
T3,	Wood vinegar application	Wood vinegar application

Throughout the crop season, the plants were checked for symptoms of sunflower charcoal-rot and maize late-wilt, and the number of infected plants (dead plants) in each plot was counted and expressed as a percentage of disease incidences. Sunflower plant heads from each plot were manually harvested 90 days after planting, dried, and seed yield was weighed (kg). The quantitative maize yield was determined by weighing the harvested plant ears from each plot 110 days after planting, drying them, and the quantitative maize yield was calculated as the weight (kilograms) of harvested ears per plot. The yield of each crop is given in kg/plot.

### Statement on guidelines

Experimental procedures and field studies on plants comply with relevant institutional, national, and international guidelines and legislation.

### Statistical analysis

The CoStat6303Win.exe software program was used to conduct a one-way analysis of variance (ANOVA) to examine differences among treatments. Mean differences were compared using Duncan's multiple range tests, and a *P* value of 0.05 was regarded as significant. The percentage data were transformed using arcsine square root prior to data analysis. The untransformed means are included in the results.

## Data Availability

The data that support the findings of this study are available from the corresponding author upon reasonable request.
